# Photosensitized INA-Labelled protein 1 (PhIL1) is novel component of the inner membrane complex and is required for *Plasmodium* parasite development

**DOI:** 10.1038/s41598-017-15781-z

**Published:** 2017-11-14

**Authors:** Ekta Saini, Mohammad Zeeshan, Declan Brady, Rajan Pandey, Gesine Kaiser, Ludek Koreny, Pradeep Kumar, Vandana Thakur, Shreyansh Tatiya, Nicholas J. Katris, Rebecca Stanway Limenitakis, Inderjeet Kaur, Judith L. Green, Andrew R. Bottrill, David S. Guttery, Ross F. Waller, Volker Heussler, Anthony A. Holder, Asif Mohmmed, Pawan Malhotra, Rita Tewari

**Affiliations:** 1 International Centre for Genetic Engineering and Biotechnology, New Delhi, 110067 India; 2 School of Life Sciences, University of Nottingham, Nottingham, NG72UH UK; 3 Institute of Cell Biology, University of Bern, Bern, 3012 Switzerland; 4 Department of Biochemistry, University of Cambridge, Cambridge, CB2 1QW UK; 5 Francis Crick Institute, London, NW1 1AT UK; 6 Protein and Nucleic Acid Chemistry Laboratory, University of Leicester, Leicester, LE2 7LX UK; 7 Department of Cancer studies, University of Leicester, Leicester, LE2 7LX UK

## Abstract

*Plasmodium* parasites, the causative agents of malaria, possess a distinctive membranous structure of flattened alveolar vesicles supported by a proteinaceous network, and referred to as the inner membrane complex (IMC). The IMC has a role in actomyosin-mediated motility and host cell invasion. Here, we examine the location, protein interactome and function of PhIL1, an IMC-associated protein on the motile and invasive stages of both human and rodent parasites. We show that PhIL1 is located in the IMC in all three invasive (merozoite, ookinete-, and sporozoite) stages of development, as well as in the male gametocyte and locates both at the apical and basal ends of ookinete and sporozoite stages. Proteins interacting with PhIL1 were identified, showing that PhIL1 was bound to only some proteins present in the glideosome motor complex (GAP50, GAPM1–3) of both *P*. *falciparum and P*. *berghei*. Analysis of PhIL1 function using gene targeting approaches indicated that the protein is required for both asexual and sexual stages of development. In conclusion, we show that PhIL1 is required for development of all zoite stages of *Plasmodium* and it is part of a novel protein complex with an overall composition overlapping with but different to that of the glideosome.

## Introduction

Intracellular protozoan parasites such as *Plasmodium*, *Toxoplasma*, *Eimeria* and *Cryptosporidium*, belong to the eukaryotic phylum Apicomplexa, and their infections result in devastating human and livestock diseases that are of immense clinical importance. *Plasmodium*, the causative agent of malaria, has a complex life-cycle in the mosquito and vertebrate host with three different forms and dimensions of invasive stages—merozoite, ookinete and sporozoite. All three extracellular forms are highly polarised and possess an apical complex at the anterior end of the cell, which is implicated in both motility and invasion of host cells and tissue. The motility of these invasive stages is powered by an actomyosin motor termed the glideosome^1^, which resides within the pellicle, tethered to the inner membrane complex (IMC) and located directly beneath the plasma membrane. The IMC is composed of flattened membrane cisternae or alveolar vesicles, whose cytoplasmic face is connected to subpellicular microtubules and a subpellicular protein network (SPN). The SPN is composed of several putative structural alveolin (or IMC) proteins as well as IMC sub-compartment proteins (ISPs), which both form broadly conserved protein families throughout Alveolate taxa^2,3^. In *Plasmodium*, the alveolins show differential expression in different invasive stages of the parasite lifecycle, namely in ookinete and sporozoite^4,5^. Several of these proteins have been implicated in cellular organisation of the non-motile stages; 17 *P*. *falciparum* (Pf) IMC proteins and two *P*. *berghei* (Pb) ISP proteins have been described as having a role in morphological differentiation during parasite development^5–8^. In addition to the structural components of the IMC, the actomyosin motor complex, or glideosome, is a central component of the invasion and motility machinery. Glideosome is comprised of various proteins including the glideosome-associated protein (GAP) 40, GAP45 and GAP50, and the class XIV myosin (Myo) A and its light chains, which are part of the outer IMC and anchored by GAP50 that is part of an integral membrane complex. Pf glideosome-associated proteins with multiple membrane spans (GAPMs) 1 to 3 also localise to the IMC and form part of the glideosome^1^. Despite these known IMC-associated protein families and complexes, it is clear that we have limited knowledge of how the shape, integrity and development of the IMC and cell pellicle are formed and controlled.

PhIL1 was identified in *T*. *gondii* (Tg) through photosensitized labelling of parasites with photoactivatable compound 5-[^125^I]iodonaphthalene-1-azide (INA) to target non GPI anchored membrane embedded protein in the pellicle^9^. PhIL1 was shown to be an integral membrane protein of the IMC, which is also associated with the cytoskeleton. Hence, it could function in mediating associations between the cytoskeleton and the IMC. PhIL1 in *T*. *gondii* is distributed over much of the IMC of the cell, but is excluded from the apical tip. It is, thus unlikely to be associated with the conoid, a conical structural element of the apical complex found throughout Apicomplexa, although not evident in some taxa including *Plasmodium* ^10^. Antibodies raised to TgPhIL1 recognised a ~25 kDa protein in *P*. *falciparum* lysates, suggesting that an orthologue might be present in this parasite^9^; however, no further information is available on the location or function of this protein in *Plasmodium*.

Here, we examine the cellular location and function of PhIL1 during the life-cycle of the human parasite *P*. *falciparum* and the rodent parasite *P*. *berghei* by generating PhIL1-GFP expressing transgenic lines. Live cell imaging and interactome analysis showed that PhIL1 has an IMC-like location during all three invasive stages, the merozoite, ookinete and sporozoite, but was most concentrated at both the apical and basal ends of the parasite in each stage. PhIL1 expression was also observed in the sexual stages, especially during male gametogenesis but was absent in female gametocytes. In genetic studies using a conditional knockdown strategy, we show that PhIL1 is likely essential for blood stage schizogony and a partial reduction in PhIL1 level during the sexual stages is tolerated, although with a delay to parasite development; this genetic modification has no effect on liver stages. In a parasite line in which PhIL1 was tagged with GFP, a novel interacting protein complex was identified; this complex contained GAP50 and GAPM1–3 but is distinct from the glideosome.

## Results

### PhIL1 has a peripheral localisation in both asexual and sexual stages of the *Plasmodium* life-cycle

Examination of the genomic databases for apicomplexan parasites indicated that the PhIL1 gene is conserved across species. Therefore, to investigate the temporal and spatial pattern of *Plasmodium* PhIL1 expression and localisation in *Plasmodium*, we generated parasite lines expressing C-terminal GFP-tagged PhIL1 in both *P*. *falciparum* (PF3D7_0109000) and *P*. *berghei* (PBANKA_0204600). The *P*. *falciparum* transgenic line allowed examination of mainly asexual blood stage biology, and PhIL1 expression during the different developmental stages in both mammalian and insect vector hosts was examined using the *P*. *berghei* transgenic line.

**Figure 1:**
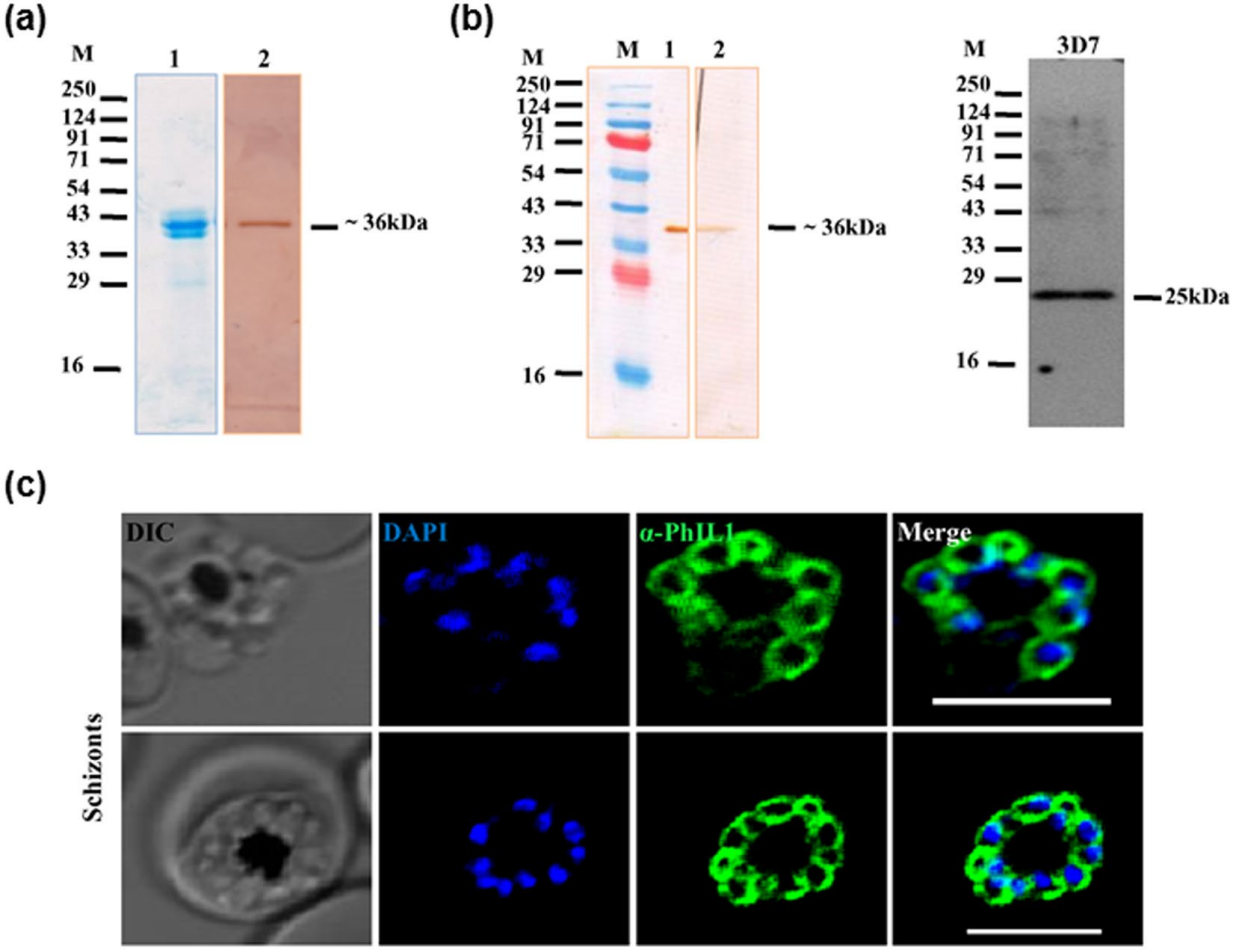
Purified antisera from recombinant PhIL1 shows specific protein bands in *P*. *falciparum* 3D7 lysates. (**a**) *Plasmodium falciparum* PhIL1 was expressed in *E*. *coli* and purified using Ni^2+^-NTA chromatography. The purified protein was analysed by SDS PAGE, stained with Coomassie Brilliant Blue (lane 1) and probed using anti-His HRP conjugated antibody (lane 2). This protein was further used to generate PhIL1 antisera in laboratory mice and rabbit. (**b**) To check the specificity of the raised PfPhIL1 antisera, the serum was used to probe recombinant protein (lane 1: rabbit antiserum, lane 2: mouse antiserum) as well as detection of PhIL1 in 3D7 parasite lysate. (**c**) Indirect immunofluorescence assay showing localisation of PfPhIL1 in the parasite, using rabbit α-PhIL1 antiserum. Scale bar = 5 μm.

Additionally, we expressed PfPhIL1 in an *E*.*coli* expression system and purified the recombinant PhIL1 protein to near homogeneity (Fig. 1a). PfPhIL1 antibodies were raised both in rabbit and mouse using the recombinant PfPhIL1. These anti-PfPhIL1 antibodies were specific as they recognized the recombinant protein as well as a specific band in lysates of 3D7 parasites by western blot analysis (Fig. 1b).

Rabbit antibody was used for IFA in 3D7 parasites, while PhIL1 antiserum was used for co-staining with GFP parasites as described below. In an indirect immunofluorescence assay of *P*. *falciparum* asexual blood stages, PhIL1 localised to the parasite periphery, consistent with an association with the IMC as seen for PhIL1 in *T*. *gondii* (Fig. 1c). We also expressed PfPhIL1 as a GFP fusion protein to allow pulldown of PfPhIL1 interacting proteins (see below). PfPhIL1-GFP was expressed from an episome maintained with blasticidin selection and using the *hsp86* promoter (Supplementary Fig. S1A) and transfected in 3D7 parasites to generate a line expressing PfPhIL1-GFP (Supplementary Fig. S1B). Antibodies specific for PfPhIL1 recognised two bands in PhIL1-GFP parasite lysates, one corresponding to PhIL1-GFP and the other to the native PhIL1. The ∼50 kDa band was recognised by the GFP-specific antiserum (Supplementary Fig. S1C). Transgenic parasites expressing PhIL1-GFP showed the same pattern of location as the PhIL1 antibody in wild type cells, and a complete co-localisation of GFP and PhIL1 was observed when probed by immunofluorescence with the two antibodies (Supplementary Fig. S1D), indicating correct subcellular targeting of PhIL1-GFP.

**Figure 2:**
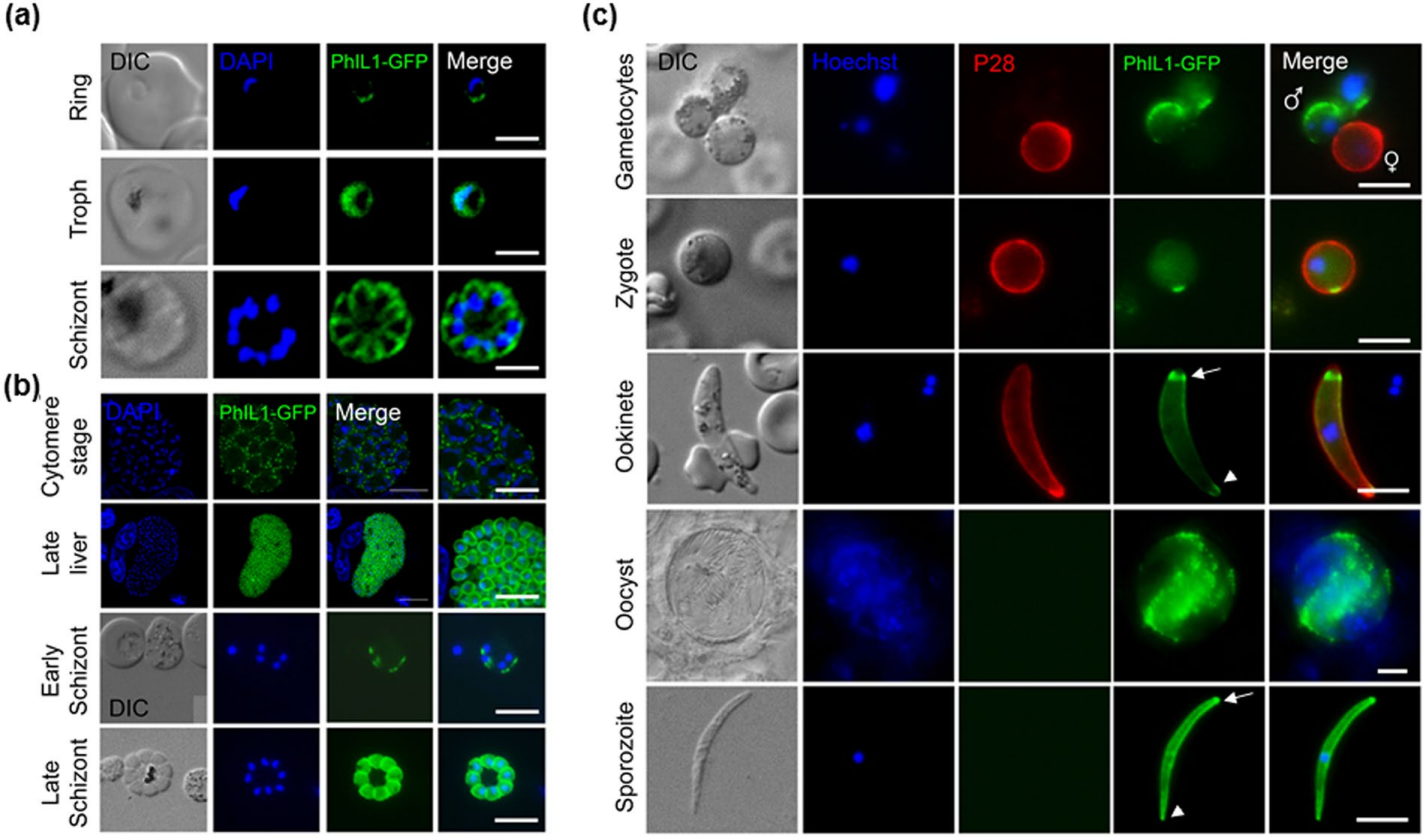
Peripheral localisation of PhIL1 during various stages of the *Plasmodium* life cycle as detected by live cell imaging. (**a**) PhIL1-GFP expression pattern in ring, trophozoite and schizont stages of *P*. *falciparum* (**b**) Localization of PhIL1-GFP in early and late stages of liver and blood stage schizogony in *P*. *berghei*. (**c**) PhIL1-GFP expression in gametocytes, zygote, ookinete, oocyst and sporozoite stages of *P*. *berghei*. 13.1, a cy3-conjugated antibody which recognises P28 on the surface of activated females, zygotes, and ookinetes was used with the sexual stages. Arrow shows apical end and arrow head the basal end of the parasite. Scale bar = 5 μm.

In *P*. *berghei*, PhIL1 expression and localisation was studied using a C-terminal GFP-tagged fusion protein created by single crossover recombination at the 3’ end of the endogenous *PhIL1* locus. Correct integration was confirmed by PCR analysis of genomic DNA using locus-specific diagnostic primers. Protein expression and size (~54 kDa) was confirmed by Western blot analysis of schizont lysates using the GFP-specific antibody (Supplementary Fig. S2). Using live cell imaging, PhIL1-GFP expression in early schizonts of *P*. *berghei* and cytomere liver stage parasites showed stronger expression of PhIL-GFP in a localisation typical of IMC formation (Fig. 2). The peripheral localisation was most evident during late schizogony in both liver (at 54 hr post infection) and blood stages as well as in the other extracellular motile invasive stages, the ookinete and sporozoite (Fig. 2b and c). In ookinetes and sporozoites a striking accumulation of PhIL1 at both apical and basal ends of the parasite was observed. Expression and peripheral localisation of PhIL1 were also observed in the activated male gametocyte, whereas no protein was observed in the female gametocyte (Fig. 2). These data suggest that PhIL1 is likely to be an IMC protein present at various stages and morphological forms of parasite development.

### PhIL1 is localised at both the apical and basal end during ookinete development and forms a ring-like structure in both ookinetes and sporozoites

**Figure 3:**
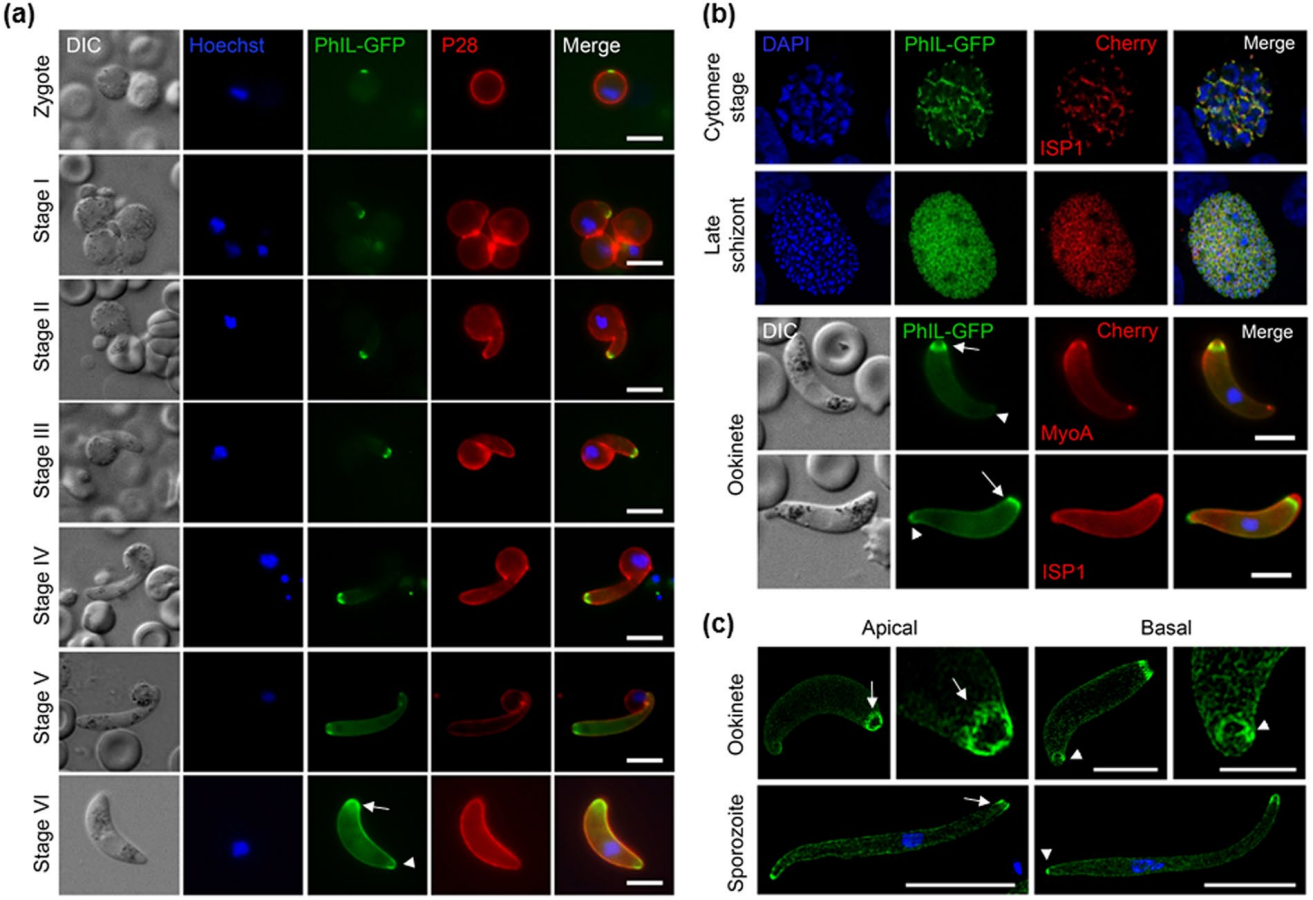
PhIL1 localisation during ookinete development, liver schizont and in sporozoites. (**a**) Expression of PhIL1-GFP during different stages (I-VI) of ookinete development. (**b**) Co-staining of ISP1 and MyoA protein with PhIL1 in early and late hepatocyte schizont stages and ookinetes. (**c**) PhIL1-GFP locates as a ring at the apical end of the ookinete and sporozoite stages, observed by super resolution microscopy, and ring or cap at the basal end. Arrow shows apical end and arrow head the basal end of the parasite. The refractive index of the immersion oil (Cargille) was adjusted to minimize spherical aberrations and sections were acquired at 0.125 μm z steps. Scale bar = 5 μm.

Since there was no PhIL1 expression in female gametocytes, but strong expression in differentiated ookinetes at both the apical and basal end, we further studied the temporal profile of PhIL1 expression during ookinete differentiation from the zygote to ascertain the potential participation of this protein in the establishment of polarity. We used live cell imaging of PhIL1-GFP localisation throughout stages I to VI of ookinete development^11,12^. Two hours post-fertilisation (stage I), a peripheral crescent-shaped protein localisation was observed in the zygote. With the initial protrusion of the membrane during stage I and II of zygote development, PhIL1–GFP was localised at the tip of this protrusion and this apical location persisted throughout early development and differentiation. The basal localisation was only observed at stage V, 15 to 18 hours post fertilisation, when ookinete development had progressed to the point when the nucleus had commenced migration to the centre of the ookinete body (Fig. 3a).

To investigate further the location of PhIL1 relative to other apical and uniform IMC markers, we crossed PhIL1-GFP parasites with lines expressing ISP1-Cherry (uniform IMC marker) and MyoA-Cherry (apical marker) lines. During liver stage development, PhIL1-GFP co-localized with MyoA-mCherry showing a similar overall distribution (Fig. 3b). In ookinetes, while a substantial portion of the peripheral weak PhIL1 staining colocalised with ISP1, the very apical end of the ISP1 staining was devoid of PhIL1 staining. MyoA-Cherry also displays a subapical ring-shaped location, but this also appeared as slightly apical to the strong sub-apical staining shown by PhIL1 (Fig. 3b). Therefore PhIL1 displays a distribution in ookinetes unlike that of either ISP1 or MyoA.

To achieve greater resolution of the PhIL1-GFP location, we used 3D-SIM super resolution fluorescence microscopy. This revealed that the distal concentration of PhIL1 forms a discrete ring in both ookinetes and sporozoites. In ookinetes the apical ring is in a sub-apical position forming a band or collar (Fig. 3c). This is different from the very tight band at the apical tip of the ookinete formed by *Plasmodium* SAS6L^12^. A similar broad band of PhIL1 at the apex is evident in sporozoites indicating a similar structure (Fig. 3c). At the posterior end of ookinetes the accumulation of PhIL1 sometimes also appears as a ring, although the shape of this structure is less consistent. In sporozoites it is unclear whether the strong posterior PhIL1 signal is a cap or ring of diameter below the resolution limit of 3D-SIM (Fig. 3c). These data indicate a previously undescribed pattern of protein locations in *Plasmodium*. The apical and basal patterns of PhIL1 location, including a weaker general pellicular presence, is very similar to that seen in *Toxoplasma* tachyzoites, although the presence of rings was beyond the limits of resolution of the microscopy used in that study^9^.

### PhIL1 is part of a novel protein complex that includes GAP50 but is different from the glideosome

**Figure 4:**
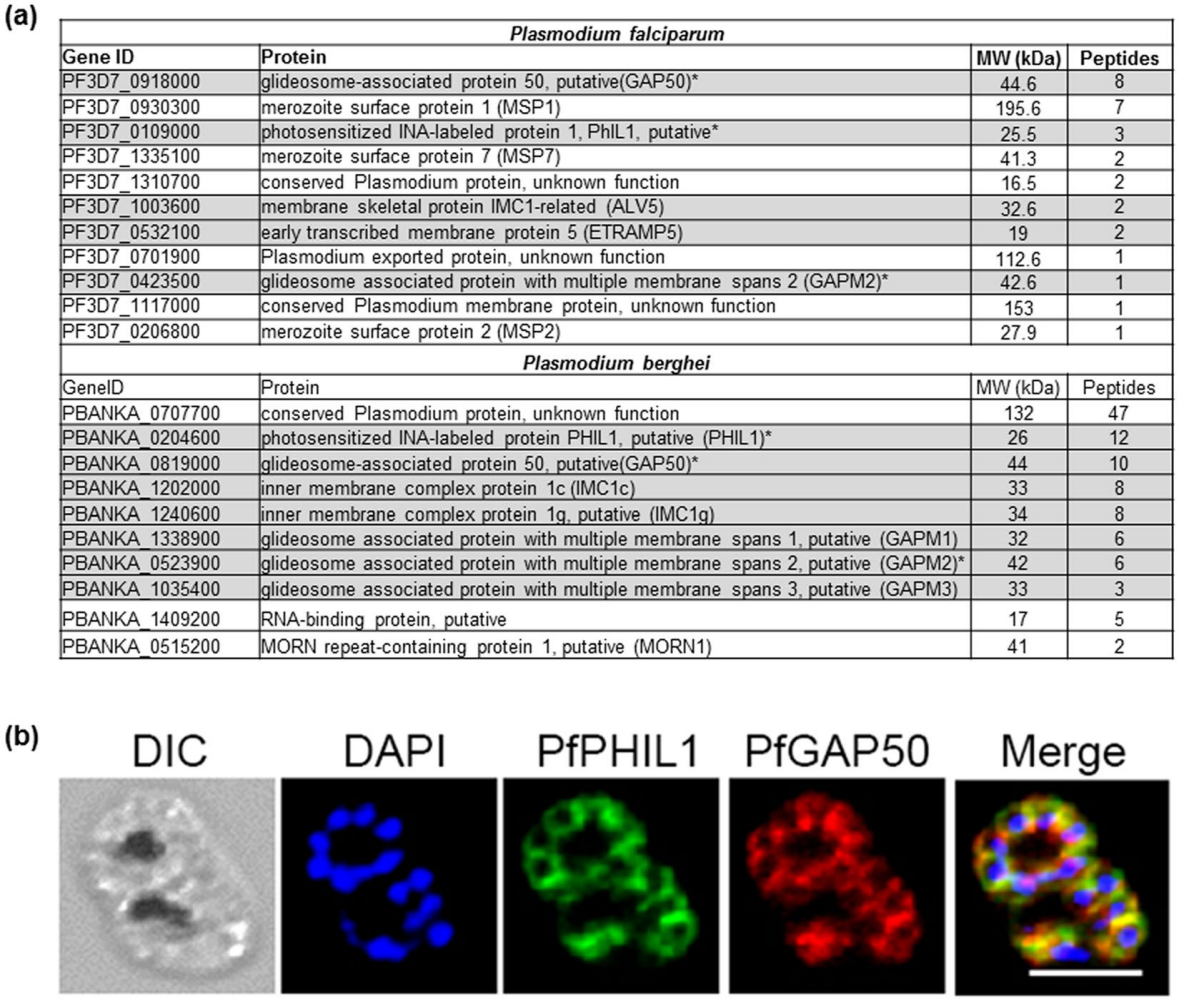
PhIL1 forms a novel protein complex, which includes GAP50 but is different from the glideosome. (**a**) List of proteins pulled down by GFP- Trap beads from lysates of PhIL1-GFP parasites from *P*. *falciparum* and *P*. *berghei*. IMC associated proteins are highlighted. *Denotes protein detected in both parasites by pulldown. The lists shown here are cumulative of three independent biological replicates and precipitated only in PhIL1-GFP lines. (**b**) Co-localization of PhIL1-GFP and GAP50 in *P*. *falciparum* blood-stage schizont. PfPhIL1 and PfGAP50 co-localise in IMC with a Pearson’s correlation coefficient of 0.78. Scale bar = 5 μm.

Having identified that PhIL1 is likely associated with the IMC, we examined the PhIL1-GFP interactome using a GFP-pulldown assay. PhIL1-GFP was isolated from cell lysates together with any interacting partners using GFP-Trap beads from both *P*. *berghei* and *P*. *falciparum* transgenic parasites. Bound proteins were then digested with trypsin and the released peptides were analysed by mass spectrometry to identify any interacting partners. Three sets of pulldown experiments recovered various IMC proteins that were co-precipitated with PhIL1 including the glideosomal proteins GAP50 and GAPM1, −2 and −3. A member of the alveolin/ IMC protein family was present in each precipitate, together with the previously uncharacterized orthologous proteins coded by PBANKA_1409200 and PF3D7_1310700. Other proteins of the glideosome complex, however, such as GAP40, GAP45, MyoA, or myosin tail interacting protein (MTIP) were not detected (Fig. 4a). As controls we used lysates of *P. falciparum* 3D7 and a line of *P*. *berghei* ubiquitously expressing GFP^13^ and the GFP-Trap beads did not precipitate any of these proteins from lysates. To validate the colocation of these interacting partners, we performed immunofluorescence analysis of *P*. *falciparum* cells stained with both PfPhIL1 and PfGAP50 antibodies. The PhIL1 and GAP50 signals significantly co-localised (Fig. 4b). Together, these results confirm the association of PhIL1 with known components of the IMC, and in particular interactions with select, but not all, components of the glideosome.

### PhIL 1 is likely essential for asexual blood stage development and it also has a role in other life cycle stages

**Figure 5:**
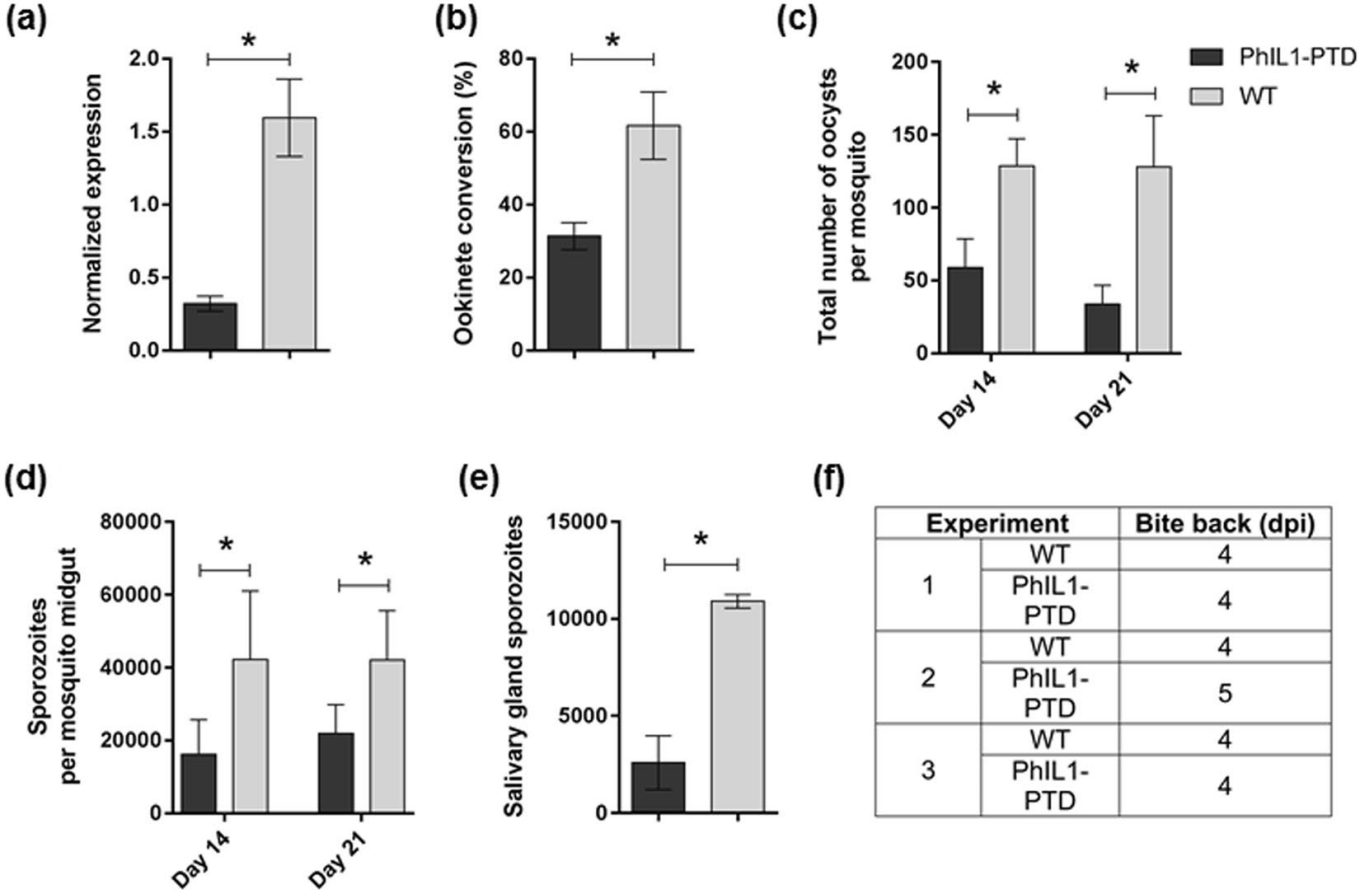
PhIL1 knockdown suggests its role at various stages of the parasite life cycle. (**a**) Conditional knock down, shows a significant reduction (60–70%) of PhIL1-PTD transcript in comparison to wild type in gametocytes of *P*. *berghei*. Three technical replicates and three biological replicates were performed for each assayed gene. The *hsp70* (PBANKA_081890) and *arginyl-t RNA synthetase* (PBANKA_143420) genes were used as endogenous control reference genes. (**b**) Ookinete conversion shows a significant decrease in the percentage of ookinetes in triplicate experiments. (**c**) The number of oocysts in the mosquito gut was also reduced in PhIL1-PTD parasite samples 14 and 21-days post infection (dpi). The infection rate of >70% was observed for both PhIL1-PTD and WT parasites in triplicate experiments. (**d**) Sporozoite number per mosquito for 14 and 21 dpi in PhIL1-PTD parasites was comparatively low in midgut oocysts in comparison to WT. (**e**) Sporozoite numbers in salivary glands on 21 dpi were also decreased in PhIL1-PTD parasites comparing to WT but, (**f**) bite-back experiments showed successful transmission of both PhIL1-PTD and WT parasites from mosquito to mouse. The prepatent period was 4–5 days for both WT and PhIL1-PTD parasites. Bar chart shows mean ± SEM for three independent experiments. *P value was < 0.05.

To study the function of PhIL1 throughout the *P*. *berghei* life cycle, we first attempted to delete the gene in the asexual blood stage using double homologous recombination, but were unable to generate a knockout despite several attempts (Supplementary Fig. S3A). This is consistent with the recent *P*. *berghei* functional screen (PlasmoGEM) that found the gene is essential^14,15^. To test the role of PhIL1 in other life cycle stages, we used a conditional knock down approach by placing the endogenous *PhIL1* locus under the control of the *ama1* promoter that is expressed in the asexual blood stage and down regulated in gametocyte and ookinete stages of parasite development. Transgenic parasites were generated using double homologous recombination to insert the promoter upstream of PhIL1 as described in Supplementary Fig. S3B. Integration of the *ama1* promoter upstream of the *PhIL1* locus was confirmed by diagnostic PCR (Supplementary Fig. S3C). Transcript analysis of these transgenic parasites (henceforth termed PhIL1-PTD, promoter trap double homologus) by qPCR revealed a substantial decrease in PhIL1 transcription in PhIL1-PTD gametocytes in comparison to wild type gametocytes (Fig. 5a). Phenotypic analysis was then carried out at various developmental stages in the PhIL1-PTD mutant in comparison to wild type control parasites. Male gametogenesis, as assessed by exflagellation, showed no significant change when compared to that in the wild-type parasite (data not shown). However, following fertilisation and zygote formation there was a 40–50% reduction in ookinete conversion in the PhIL1-PTD parasites, compared to wild-type (Fig. 5b). When *A*. *stephensi* mosquitoes were fed on mice infected with PhIL1-PTD parasites the number of oocysts was reduced to 40–50% in PhIL1 PTD parasites on day 14 and 21 post infection in comparison to WT parasites (Fig. 5c). However, the size of oocysts was similar in both WT and PhIL1-PTD parasites. The number of released sporozoites also decreased to 40–50% on days 14 and 21 post infection in the parasites (Fig. 5d), but no morphological differences were observed in the PhIL1-PTD sporozoites. When infected mosquitoes were used for bite back experiments to ascertain sporozoite infectivity, blood stage infection was observed at day 4 in naïve mice for both wild type and PhIL1-PTD parasites.

Overall, the analysis of the knockdown of PhIL1 transcription showed that depletion of PhIL1 transcript resulted in a clear phenotype of a decrease in ookinete conversion followed by a corresponding reduction in the number of oocysts and sporozoites. Some parasites could still complete the life cycle, however, it is unknown what the effect of complete loss of PhIL1 would be in these stages, and a more severe phenotype is possible given the inability to generate a gene knockout in the asexual blood-stage.

## Discussion

A unifying feature of apicomplexan parasites is that all invasive stages possess a surface pellicle and highly polarised cellular organisation with the apical complex at the anterior end of the cell implicated in motility and invasion of host cells/tissue^10^. Motility in these invasive stages is powered by an actomyosin motor termed the glideosome, which resides within the pellicle located directly beneath the surface membrane, and tethered to the IMC. Several recent studies have identified proteins associated with the IMC and provided insights into the organisation and role of this structure^1,16–18^.

PhIL1 was originally discovered as an IMC-associated protein in tachyzoites of *Toxoplasma* ^9^. Deletion of the gene showed only a very mild growth phenotype, including a reduced but not abolished parasite load in animal models, and a subtle morphological change of slightly shorter and wider parasites^19^. No loss of parasite motility was seen, although the trajectory of 3D gliding patterns did change with the cell morphology^19,20^. The presence, location, importance or function of a PhIL1 homolgoue in *Plasmodium* in invasive and/or sexual stages have not been previously investigated. We examined the localisation of PhIL1 using two species: *P*. *falciparum* and *P*. *berghei*, as the latter allows study of the sexual, sporogonic and liver stages and therefore the entire life cycle. By using parasites expressing PhIL1 tagged with GFP, we show that the protein exhibits an IMC-like localisation during liver and blood stage schizogony. This localisation was confirmed in *P*. *falciparum* schizonts using immunofluorescence with antibodies to PhIL1 and GAP50, a known component of the IMC. The protein was also detectable in the ring and trophozoite forms of the asexual blood-stage, which has not been observed for other IMC associated proteins studied to date^5–8,21^, and the function of PhIL1 in these stages is therefore not clear, which leaves an interesting stone unturned for further studies. Surprisingly whilst PhIL1 is expressed in activated male gametocytes it is not detected in female gametocytes, suggesting that the gene may be translationally repressed in females^22,23^. The early initial expression and localisation of PhIL1 in zygotes at 2 hours post-fertilisation supports this hypothesis. The PhIL1 gene has also been described as one of the target genes for the AP2-O transcription factor^5^.

The timing of the establishment of the PhIL1 apical signal during ookinete development differs from that of other apical IMC associated molecules like ISP1, which is detected before fertilization in early female gametocyte development^8^ or of other apical proteins such as SAS6L and Myosin B, which are detected after 4 hours post fertilisation or at a much later stage of ookinete development, respectively^7,12,24^. Therefore, PhIL1 has a spatial and temporal profile very different to that of many other IMC-associated proteins and proteins implicated in the apical complex. PhIL1 is unusual compared to known IMC-associated proteins in that it has both apical and basal polarity in the ookinete and sporozoite stages. The developmental pattern of PhIL1 during ookinete differentiation suggests that basal PhIL1 is organised after apical polarity during ookinete development. Very few basal localised proteins have been described in invasive stages in *Plasmodium*. Recently a transmembrane protein localising at the basal rim of nascent IMC was reported in blood stage parasites^6^. While many apically localised protein have been reported both in *Toxoplasma* and *Plasmodium*, very few have been characterised during ookinete or sporozoite stages^5–8,12,24–26^.

The super resolution microscopy studies described here indicate that PhIL1 is associated with a ring structure at the apical end, and might also form a ring at the basal end of the parasite. Its location is distinct from that of SAS6L, the other ring protein studied in *Plasmodium* ^12^. Many IMC, apical complex and ring proteins at the conoid (in *Toxoplasma*) are implicated in establishing apical polarity in *Plasmodium* and *Toxoplasma* ^5,7,8,25,27,28^. Since the sexual stages are difficult to obtain for many other apicomplexan parasites, the studies here provide important evidence for the role of PhIL1 during sexual and sporogony stages.

IMC proteins have been implicated as anchors for the actomyosin machinery used by the parasite for motility and invasion of host cells^1,29^. To obtain insight into the interactions of PhIL1 with other proteins, we identified binding partners following immuno-purification. Our analysis showed that PhIL1-GFP interacts with glideosome-associated proteins including GAP50, and GAPM1, −2 and −3 but it does not bind MyoA, MTIP or GAP45, proteins which are also crucial components of the glideosome^1,29^. These results suggest that PhIL1 forms a novel protein complex with alveolins and components that overlap with those in the known glideosome complex, including GAP50. It may be that GAPM1 to −3 are proteins which span both the outer and inner side of the IMC and hence may be components of complexes on either side of the IMC. Our IFA studies showed that PhIL1 colocalises with GAP50, while it has a different location to that of MyoA. Since GAP50 is both a lumenal and transmembrane protein it is possible that PhIL1 interacts with GAP50 on the inner membrane of the IMC and GAP50 interacts on the outer side with the myosin machinery like GAP45, GAP40, MyoA and MTIP. GAP50 has been shown to be part of the gametocyte membrane complex in *P*. *falciparum* ^30^. This suggests that PhIL1 and GAP50 may form another complex distinct from the glideosome, and which may be important in both sexual and asexual stages. The presence of MORN1 in the *P*. *berghei* interactome also suggests that PhIL1 may be part of the cell division machinery and also the part of the basal end of the parasite as seen in *Toxoplasma* ^31–33^. A recent study has shown that a pellicle protein, BTP1 is localised to the basal rim of the IMC during *P*. *falciparum* schizogony and co-localises with MORN1^6^. The presence of MORN1 in our interactome data suggests that PhIL1 may also be part of the basal complex in *P*. *berghei*. To our knowledge this is the first description of a basal protein in ookinete and sporozoite stages of *Plasmodium*.

Our inability to knockout *P*. *berghei* PhIL1 by double homologous recombination indicates that the gene is likely essential for blood stage replication, a conclusion that is consistent with the recent functional profiling of the *Plasmodium* genome^14^. This suggests a greater susceptibility of *Plasmodium* to PhIL1 loss than seen in *Toxoplasma*. One possibility for this is that the *in vitro* system exposes the parasite to different stresses, or that there is greater redundancy of molecules maintaining IMC strength, integrity or functions such as motility and invasion in *Toxoplasma*. This has been recently suggested for the difference in gene essentiality between *Plasmodium* and *Toxoplasma* in genome-wide function screens in Toxoplasma and *Plasmodium* ^14,34^.

The conditional knockdown of PhIL1 expression using the *ama1* promoter is an approach that has been utilised in previous studies of MyoA, MTIP and other motor complex genes^35^. The strategy produced viable parasites for MyoA and MTIP knockdowns suggesting that expression of these genes is not essential in ring and trophozoite stage parasites, since AMA1 is mostly expressed during late stages of schizogony. We could not completely knockdown PhIL1 transcription at gametocyte and ookinete stages of the life cycle suggesting that even partial expression of PhIL1 at these various stages is sufficient for the parasite to complete development. This could explain why we observed a significant decrease, but not complete ablation of exflagellation during male gametogenesis and of ookinete conversion in PhIL1-PTD parasites. This observation also correlates well with the fact that PhIL1-GFP expression was detected during male gametogenesis and ookinete development. The specific location of PhIL1-GFP in the male gametocyte and its role during male exflagellation and ookinete differentiation shows that IMC proteins are not only relevant for invasive stages but are involved in gametocyte/zygote differentiation. While there is no apical polar ring in gametocytes, PhIL1 might contribute to the IMC-like complex of the male gametocyte. IMC proteins have been shown to be present at the periphery of mature *P*. *falciparum* gametocytes^36^, however PhIL 1 was not shown to be part of this complex^36^. It will be interesting to identify the proteins interacting with PhIL1 during male gametogenesis and ookinete in future studies. Given that use of the AMA1 promoter only partially reduced PhIL1 expression, and that this allowed the parasite to complete its life cycle, albeit with reduced efficiency, it may be useful to use other gene promoters to control expression. *clag1*, which is active in ring, trophozoite and schizont stages and tightly regulated throughout the other life-cycle stages, offers one such option, and this promoter could be used to test whether a stronger transcription knockdown has more severe effects, as predicted by the attempts at gene knockout. This approach has been used to study the function of *cdpk1* in ookinete development^35^.

Overall, we have demonstrated that PhIL1 is an IMC-associated protein that is not only present in motile and invasive stages but also in sexual stages, especially during male gametogenesis and ookinete differentiation. It defines both apical and basal polarity in the parasite and is essential in the life-cycle. We show that it forms a novel protein complex that shares some similar composition, but also differences from the glideosome. Other components of this complex will be studied in the future to investigate their function.

## Material and Methods

### Ethics statement

The animal work performed in this study was approved by the Institutional Animals Ethics Committee of ICGEB (IAEC-ICGEB) under the approval number ICGEB/AH/2015/01/MAL-74 and by the United Kingdom Home Office with the project licence number 30/3248. Rotary blood bank provided human red blood cells. Work was carried out according to the guidelines of IAEC-ICGEB and in accordance with the United Kingdom ‘Animals (Scientific Procedures) Act 1986’ and in compliance with ‘European Directive 86/609/EEC’ for the protection of animals used for experimental purposes.

Tuck’s Original (TO) (Harlan) outbred mice were used for all experiments of *Plasmodium berghei*. New Zealand White rabbits and BALB/c mice were used for raising the PhIL1 antibodies.

### For Plasmodium falciparum

#### Recombinant protein expression and generation of immune sera

The entire coding region of PfPhIL1 (PF3D7_0109000) was amplified using the primer sets: PHIL1_F and PhIL1_R (Supplementary Table 1), subcloned into pET28a expression vector (Invitrogen Life Technologies), expressed in BL21 (DE3) cells, and induced with 1 mM IPTG. The recombinant histidine tagged PhIL1 protein was purified by Ni^2+^-NTA affinity chromatography. The purified recombinant protein was analysed on 12% SDS-PAGE and further confirmed with western blot using anti-His6 monoclonal antibody (Sigma-Aldrich). Protein specific antibodies were raised in rabbit and mice following standard procedures.

#### Maintenance of *P*. *falciparum* cultures and transfection

The *P*. *falciparum* parasite line 3D7 was maintained as described previously^37,38^. To generate a transfection vector construct, the entire open reading frame of PfPhIL1 was amplified using PfPhIL1-F and PfPhIL1-R primer set (Supplementary Table 1), and cloned into the pSSPF2 vector^39^ to create a GFP- PfPhIL1 protein under the control of *hsp86* promoter. *P*. *falciparum* 3D7 ring stage parasites were transfected with 100 μg of plasmid DNA by electroporation (310 V, 950 μF)^40^ and the transfected parasites were selected using 2.5 nM blasticidin. Expression of the PfPhIL1-GFP fusion protein in transgenic *P*. *falciparum* blood stage parasites was examined by western blotting. Protein bands were visualized using an ECL kit (Thermo Scientific, USA).

Fluorescence microscopy: To visualize GFP expression, the transgenic parasite suspension was incubated with DAPI (2ng/ml) in PBS at RT for 10 min and observed on a Nikon A1 Confocal Microscope (Nikon Corporation, Japan).

#### Indirect Immunofluorescence Assay

Parasites were fixed with solution containing 4% paraformaldehyde (PFA) and 0.0075% glutaraldehyde in PBS. Parasites were then subjected to permeabilization with 0.1% Triton X-100 followed by blocking with 5% FBS. After blocking, parasites were probed with primary antibody for 1 h, followed by secondary antibody for 1 h. The parasites were then incubated with DAPI and imaged using a Nikon A1-R confocal microscope. The images were analysed by NIS elements software (Nikon). The antibody combinations used are: 1. rabbit anti-PfPhIL (1:200) for PhIL1localisation in 3D7 parasites, 2. For confirming the GFP line, rabbit α-PfGFP (1:250) and mice anti-PfPhIL (1:100) were used, 3. For colocalisation studies, rabbit α-PfGAP50 (1:250), mice anti-PfPhIL (1:100) were used; followed by appropriate secondary antibodies: anti-mice Alexafluor 488 (1:500), anti-rabbit Alexafluor 488 (1:500) anti-rabbit Alexafluor 594 (1:500) (Invitrogen).

#### Immunoprecipitation of GFP-fusion protein and identification by mass spectrometry

The mature schizont/merozoite stage lysate was obtained and immunoprecipitation was done using GFP-Trap^®^_A Kit (Chromotek) following the manufacturer’s instructions. GFP-Trap^®^_A beads were allowed to bind to parasite lysate by tumbling the tube end-over-end. Proteins were eluted in 50 μl elution buffer, digested with trypsin and peptides were analysed by mass spectrometry^41^.

### For Plasmodium berghei

#### Generation of transgenic parasites

The oligonucleotides used to generate the mutant parasite lines can be found in Supplementary Table 1. A schematic representation of the endogenous *PhIL1* locus (PBANKA_0204600), the constructs and the recombined *PhIL1* locus can be found in Supplementary Fig. 1. For GFP-tagging of *PhIL1* by single crossover homologous recombination, a region of *PhIL1* downstream of the ATG start codon was used to generate the construct as described previously^42^.The gene deletion targeting vector for ∆*PhIL1 was* constructed using the pBS-DHFR plasmid, which contains polylinker sites flanking a *T*. *gondii dhfr/ts* expression cassette confering resistance to pyrimethamine, as described previously^43^. The conditional knockdown construct (PhIL1-PTD) was derived from *P*_*ama1*_ (*pSS368*) where *PhIL1* was placed under the control of *ama1* promoter, as described previously^35^. *P*. *berghei* ANKA line 2.34 (for GFP-tagging) or ANKA line 507cl1 expressing GFP^13^ (for gene deletion and promoter trap) parasites were transfected by electroporation.

#### Genotypic analysis of parasites

For the genotypic analyses of C-terminal GFP tagged PhIL1 parasites, a diagnostic PCR reaction was performed as outlined in Fig. S2A. Primer 1 (intT221) and primer 2 (ol492) were used to determine correct integration of the *gfp* sequence at the targeted locus. A diagnostic PCR was performed for *PhIL1* gene knockdown parasites as outlined in Fig. S3B. Primer 1 (intPTD295) and Primer 2 (5’-intPTD) were used to determine successful integration of the targeting construct at the 5’ gene locus. Primer 3 (3’-intPTD) and Primer 4 (intPTD293) were used to determine successful integration for the 3’ end of the gene locus. All the primer sequences can be found in Supplementary Table 1.

#### Phenotypic analyses

Blood containing approximately 50,000 parasites was injected intraperitoneally (i.p) into mice to initiate infections. Four to five days post infection, exflagellation and ookinete conversion were examined as described previously^42^ with a Zeiss AxioImager M2 microscope (Carl Zeiss, Inc) fitted with an AxioCam ICc1 digital camera. To analyse mosquito transmission, 30–50 *Anopheles stephensi* SD 500 mosquitoes were allowed to feed on anaesthetized, infected mice whose asexual parasitaemia had reached up to 15% and were carrying comparable numbers of gametocytes. To assess mid-gut infection, approximately 15 guts were dissected from mosquitoes on day 14 post feeding and oocysts were counted on Zeiss AxioImager M2 microscope. On day 21 post-feeding, another 20 mosquitoes were dissected and their guts and salivary glands crushed separately in a loosely fitting homogenizer to release sporozoites, which were then quantified using a haemocytometer or used for imaging. Mosquito bite back experiments were performed 21 days post-feeding using naive mice and blood smears were examined after 3–4 days.

#### Culture and gradient purification of schizonts, gametocytes and ookinetes

Blood cells obtained from infected mice (day 5 post infection) were cultured for 24 h at 37 °C (with rotation at 100 rpm) and schizonts were purified the following day on a 60% v/v NycoDenz (in PBS) gradient, (NycoDenz stock solution: 27.6% w/v NycoDenz in 5 mM Tris-HCl, pH 7.20, 3 mM KCl, 0.3 mM EDTA). Purification of gametocytes was achieved using a protocol modified from ref.^44^. On day four post-infection mice were bled by cardiac puncture into heparin and gametocytes separated from uninfected erythrocytes on a 48% (v/v) NycoDenz in coelenterazine loading buffer (CLB; PBS, 20 mM HEPES, 20 mM Glucose, 4 mM sodium bicarbonate, 1 mM EGTA, 0.1% w/v BSA, pH 7.25) gradient. Blood cells from day 5 post infection mice were placed in culture for 24 hrs at 20 °C for ookinete production and purified on a 62.5% v/v NycoDenz (in PBS) gradient.

#### Liver stage cultures

HeLa cells (European Cell Culture Collection) were grown in minimum essential medium with Earle’s salts, supplemented with 10% heat inactivated foetal calf serum, 1% penicillin/streptomycin and 1% L-glutamine (PAA Laboratories) in a humidified incubator at 37 °C with 5% CO_2_. For infection, 1 × 10^5^ HeLa cells were seeded into glass bottom dishes (MatTek). The day after seeding, salivary glands were removed from female *Anopheles stephensi* mosquitoes infected with PbPhIL1-GFP parasites and following mechanical disruption, sporozoites, in infection medium (MEM with 2.5 µg/ml Amphotericin B (PAA Laboratories), were used to infect the seeded HeLa cells. For live cell imaging, at 54 hpi Hoechst 33342 (Sigma) was added to infected cells at 1 μg/ml. Confocal live cell images of parasites were acquired using the Leica TCS SP8 confocal microscope with the Leica Application Suite X software.

#### qRT-PCR analysis

The total RNA was isolated from purified gametocyte stage of parasite using the Absolutely RNA purification kit (Stratagene). The cDNA was synthesised using an RNA-to-cDNA kit (Applied Biosystems). Gene expression was quantified by qPCR using SYBR green fast master mix (Applied Biosystems). Analysis was conducted using an Applied Biosystems 7500 fast machine with the following cycling conditions: 95 °C for 20 sec followed by 40 cycles of 95 °C for 3 sec; 60 °C for 30 sec. The primers used for qPCR can be found in Supplementary Table 1.

#### Super resolution Imaging

Purified ookinetes or salivary gland sporozoites were fixed with 2% PFA and allowed to settle onto PEI-treated No. 1.5 H coverslips (0.170 mm ± 0.005 mm) for 20 minutes, stained with DAPI, rinsed in water and mounted in Vectashield as described earlier^12^.Super-resolution images were acquired using a Deltavision OMX 3D-SIM System V3 BLAZE from Applied Precision (GE Healthcare). Imaging of each channel was done sequentially using three angles and five phase shifts of the illumination pattern as described previously^45^. Raw OMX data was reconstructed and channel registered in SoftWoRx software version 6.1.3 (Applied Precision, GE Healthcare).

#### Statistical analysis

Statistical analyses were performed using Graph Pad Prism 5 software. An unpaired t-test was conducted to examine significant differences between wild-type and mutant strains.

## Electronic supplementary material

Supplementary information
