# Supplementary material for: Photosensitized INA-Labelled protein 1 (PhIL1) is novel component of the inner membrane complex and is required for Plasmodium parasite development
Source: Sci Rep. 2017 Nov 14;7:15577. doi: 10.1038/s41598-017-15781-z (PMC5686188; doi:10.1038/s41598-017-15781-z)
Supplement: Supplementary file 1 — Supplementary information [file 41598_2017_15781_MOESM1_ESM.pdf]

Photosensitized INA-Labelled protein 1 (PhIL1) is novel component of the inner membrane complex and is required for *Plasmodium* parasite development.

Ekta Saini[1+], Mohammad Zeeshan[2+], Declan Brady[2], Rajan Pandey[1], Gesine Kaiser[3], Ludek Koreny[4], Pradeep Kumar[1], Vandana Thakur[1], Shreyansh Tatiya[1], Nicholas J. Katris[4] Rebecca Limenitakis[3], Inderjeet Kaur[1], Judith L. Green[5], Andrew R. Bottrill[6], David S. Guttery[7], Ross F. Waller[4], Volker Heussler[3], Anthony A. Holder[5], Asif Mohmmed[1], Pawan Malhotra[1*] and Rita Tewari[2*]

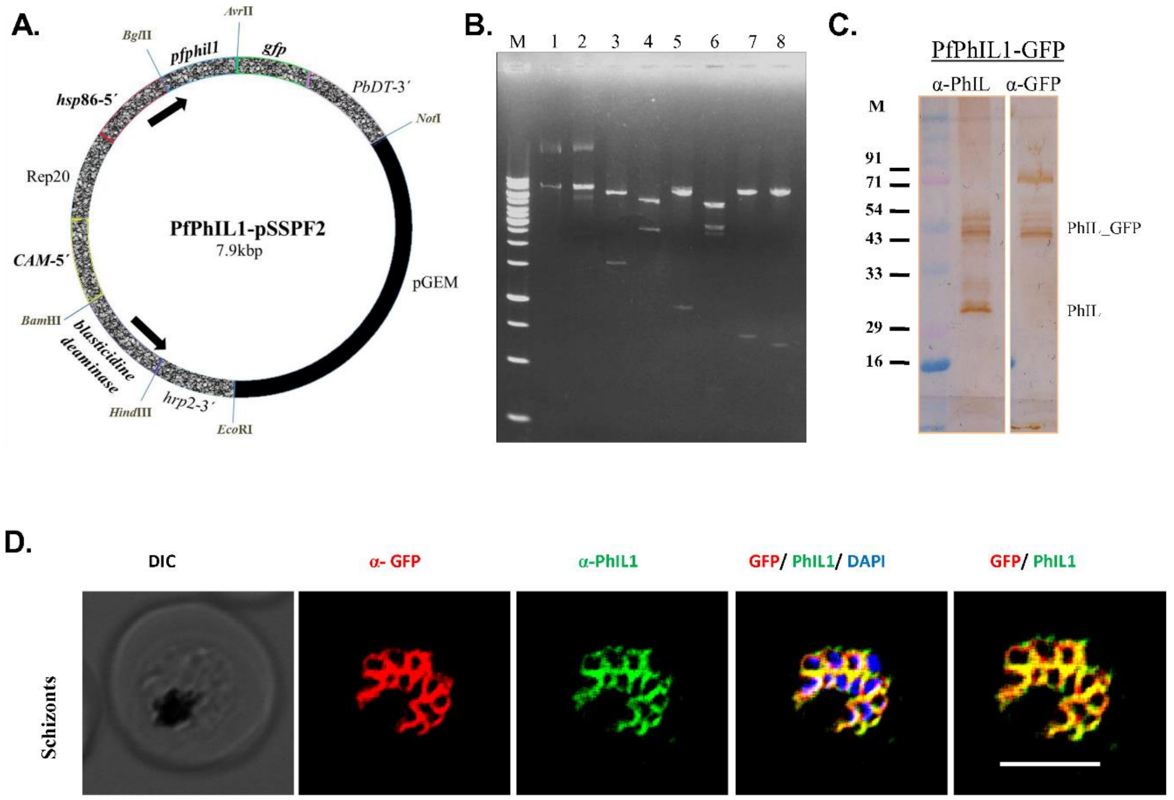

Supplementary Figure1. **Generation of PfPhIL1-GFP fusion tagging construct.** To detect expression of the PfPhIL1-GFP fusion protein in the transgenic line, parasite lysates were analysed using western blotting with anti-GFP antibody. IFA was also performed with GFP-specific and PfPhIL1-specific antibodies to check the correct targeting of the PhIL1-GFP protein. **A**. PfPhIL1 was cloned into the pSSPF2 vector using *Bgl*II and *Avr*II restriction sites to generate a C-terminal GFP fusion PfPhIL1. **B.** The construct PfPhIL1-pSSPF2 was checked for correct insertion of PhIL1 using different sets of restriction digestions. Lan**e** 1: undigested construct, lane 2: *Bgl*II/*BamH*I, lane 3: *Not*I/*Avr*II, lane 4: *EcoR*I/*Not*I for pGEM backbone, lane 5: *Bam*HI/*EcoR*I, lane 6: *BamH*I/*Hind*III for blasticidin resistance gene, lane7: AvrII/XhoI for GFP tag, lane 8: BglII/AvrII for PhIL1 gene. **C.** Western blot analysis of lysate from PfPhIL1-GFP tag line with α-GFP rabbit serum and α-PfPhIL 1 rabbit sera. **D.** Co-localisation of GFP and PhIL1 proteins using anti-GFP and PhIL1 antisera; indirect immunofluorescence assay showing co-staining of PfPhIL1-GFP fusion protein and PfPhIL1 protein with a Pearson correlation coefficient of 0.92. Scale bar = 5 μm.

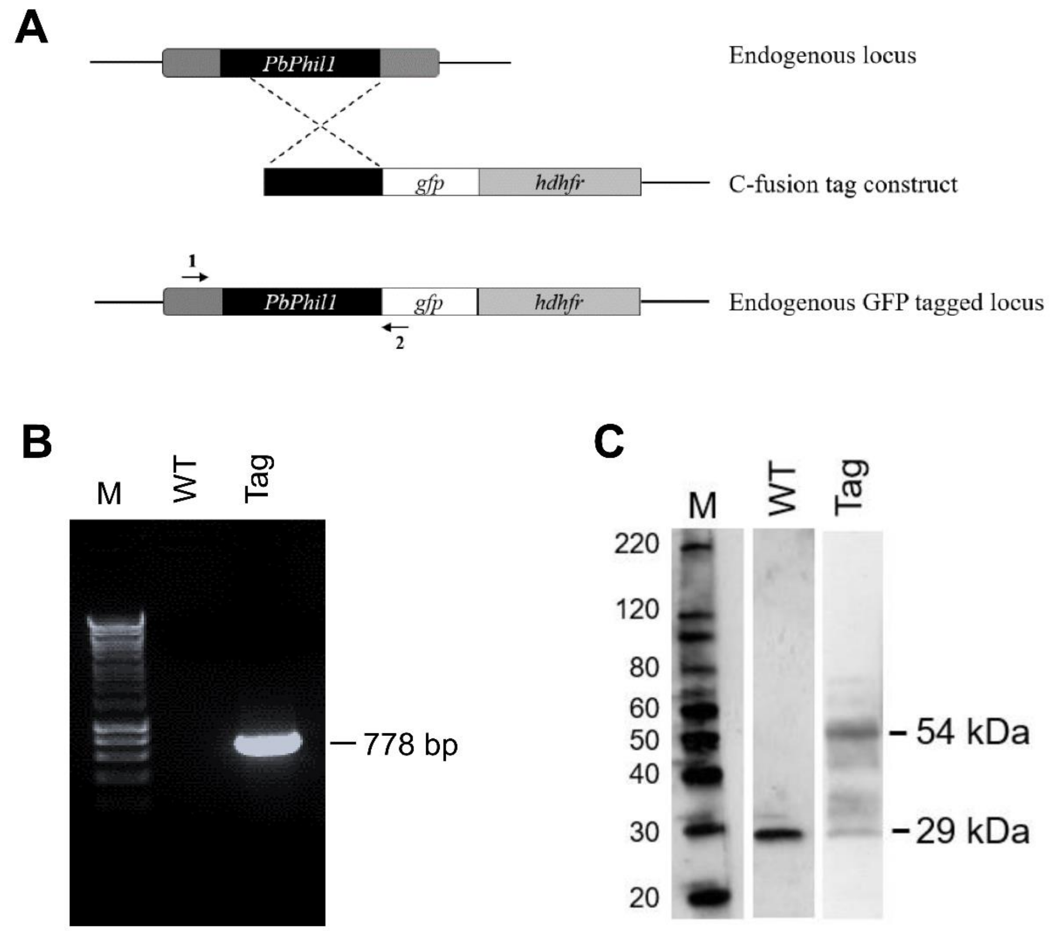

Supplementary Figure 2. **Generation and genotype analysis of PhIL-GFP in *P. berghei***

(A) Schematic representation of the endogenous *PhIL1* locus, the GFP-tagging construct and the recombined *PhIL1* locus following single homologous recombination. Arrows 1 and 2 indicate the position of PCR primers used to confirm successful integration of the construct. **(B)** Diagnostic PCR of PhIL 1-GFP and WT parasites using primers IntT221 (Arrow 1) and ol492 (Arrow 2). Integration of the PhIL1 tagging construct gives a band of 778 bp. Tag = PhIL1-GFP parasite line. **(C)** Western blot of PhIL1-GFP (54 kDa) and WT-GFP (29 kDa) protein to illustrate PhIL1-GFP in schizont stage.

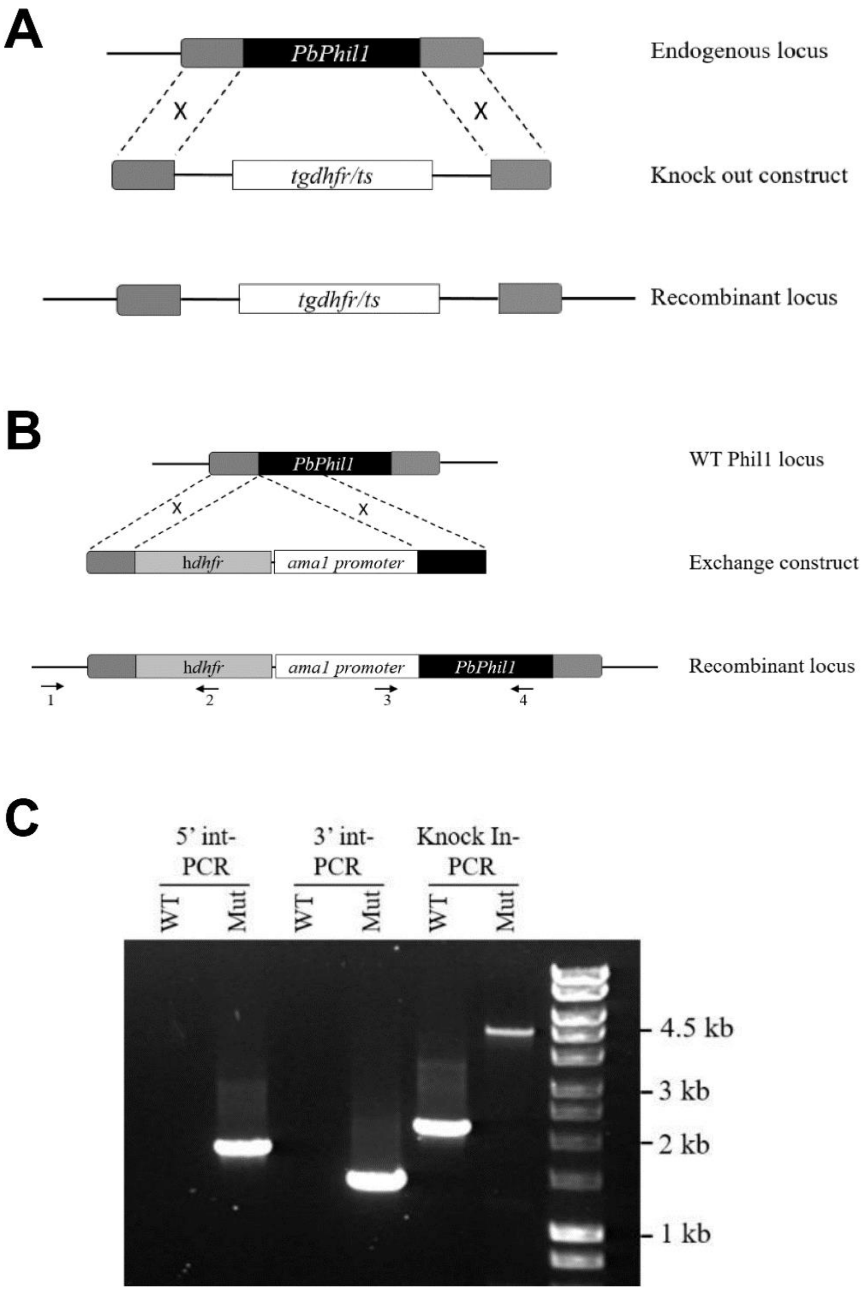

Supplementary Figure 3. Generation and genotype analysis of PhIL1 Promoter trap parasites (A) Schematic representation of the endogenous *PhIL1* locus, the targeting gene deletion construct and the recombined *PhIL1* locus following double homologous recombination. **(B)** Schematic representation of the promoter swap strategy (PhIL1-PTD, placing *PhIL-1* under the control of the *ama1* promoter) by double homologous recombination. Arrows 1 and 2 indicate the primers position used to confirm 5' integration and arrows 3 and 4 indicate the primers used for 3' integration **(C)** Integration PCR of the promotor swap

construct into the *PhIL1* locus. Primer 1 (IntPTD295) with primer 2 (5'-IntPTD) were used to determine successful integration of the selectable marker resulting in a band of 1762 bp. Primer 3 (3'-intPTama1) and primer 4 (IntPTD293) were used to determine the successful integration of *ama1* promoter resulting in a band of 1387 bp. Primer 1 (IntPTD295) and primer 4 (IntPTD293) were used to show complete knock-in of the construct with a band at 4.5kb and the absence of a band at 2.1 kb (endogenous) resulting in complete knock-in of the construct.

Supplementary Table 1. Oligonucleotides used in this study

Plasmodium falciparum		
**Name**	Sequence 5' to 3'	Notes
**Primers used for cloning in**		
**PHIL1_F**	GCCATGGGATCCATGCTTTCTTCCATATCACC	BamHI site underlined
**PhIL1_R**	GCGTCGACCTCGAGCATATCTTGGTTATAATTTTCTTG	XhoI site underlined
**Primers used for GFP tag construct**		
**PfPhiL1-F**	AGACTCTTCCTTTAGATGCTTTCTTCC	BglII site underlined
**PfPhiL1-R**	CCTAGGCATATCTTGGTTATAATTTTCTTG	AvrII site underlined
Plasmodium berghei		
**Name**	Sequence 5' to 3'	Notes
**Primers used for GFP tag construct**		
**T2211**	CCCCGGTACCCAGTATAATTTTCCCTTCCGAAC	KpnI site underlined
**T2212**	CCCCGGGCCCCATATTATCTTTAGGGCATTCTTG	ApaI site underlined
**Int221**	CAACACCGAGAAAAAGATATAGT	
**ol492**	ACGCTGAACTTGTGGCCG	
**Primers used for knockout construct**		
**N1221**	CCCCGGGCCCATTTGTAGAACTTACAACGATCAAGT	ApaI site underlined
**N1222**	GGGGAAGCTTAAATAAAAAGGTTAGTTGCTTGTTCAT	HindIII site underlined
**N1223**	CCCCGAATTCTGACATTGCTCGATTTTTGCCA	EcoRI site underlined
**N1224**	GGGGTCTAGATCAATCCTCCCAATGGGACC	XbaI site underlined
**Primers used for Promotor swap construct**		
**PTD0291**	GAGACCGCGGAGGGGAAATTGAAAAGCGTGA	SacII site underlined
**PTD0292**	GAGACTGCAGCTAAAGTTTAAAATTATTTTAGAAAATATTAATTTTTTTT	PstI site underlined
**PTD0293**	GAGACTCGAGATGCTTTTTTCAACACCGAG	XhoI site underlined
**PTD0294**	GAGAGCGGCCGCGATATCTTATGCGGATTTGTCAC	NotI site underlined
**IntPTD295**	CTTTCCAAAGTTATGAATTATACATAAC	
**5'-intPTD**	TCTACTTTATTTGCTAATTCTGG	
**3'-intPTama1**	TGTGTATATATAAGTATTGTATGGTAATTG	
**IntPTD293**	TCAATCCTCCCAATGGGACC	
**Primers used for qRT PCR**		
**hsp70 FW**	GTATTATTAATGAACCCACCGCT	PBANKA_081890
**hsp70 RV**	GAAACATCAAATGTACCACCTCC	
**arginyl-tRNA FW**	TTGATTCATGTTGGATTTGGCT	PBANKA_143420
**arginyl-tRNA RV**	ATCCTTCTTTGCCCTTTCAG	
**Phil1 FW**	GCACCTGAAATGCAAACTGGA	PBANKA_0204600
**Phil1 RV**	CCATTCTGAGTTTGCTTCACCC	
